# A Novel Fetal Movement Simulator for the Performance Evaluation of Vibration Sensors for Wearable Fetal Movement Monitors

**DOI:** 10.3390/s20216020

**Published:** 2020-10-23

**Authors:** Abhishek Kumar Ghosh, Sonny F. Burniston, Daniel Krentzel, Abhishek Roy, Adil Shoaib Sheikh, Talha Siddiq, Paula Mai Phuong Trinh, Marta Mambrilla Velazquez, Hei-Ting Vielle, Niamh C. Nowlan, Ravi Vaidyanathan

**Affiliations:** 1 Department of Mechanical Engineering, Imperial College London, London SW7 2AZ, UK; a.ghosh18@imperial.ac.uk; 2 Department of Bioengineering, Imperial College London, London SW7 2AZ, UK; sonnyburniston@yahoo.co.uk (S.F.B.); daniel.krentzel16@imperial.ac.uk (D.K.); royabhishek77@gmail.com (A.R.); adil.sheikh15@imperial.ac.uk (A.S.S.); talha.siddiq15@imperial.ac.uk (T.S.); mai.trinh16@imperial.ac.uk (P.M.P.T.); martamamvel@hotmail.com (M.M.V.); hei-ting.vielle16@imperial.ac.uk (H.-T.V.); n.nowlan@imperial.ac.uk (N.C.N.)

**Keywords:** fetal movement simulator, fetal movement monitor, maternal abdomen model, acoustic sensor, accelerometer, piezoelectric diaphragm

## Abstract

Fetal movements (FM) are an important factor in the assessment of fetal health. However, there is currently no reliable way to monitor FM outside clinical environs. While extensive research has been carried out using accelerometer-based systems to monitor FM, the desired accuracy of detection is yet to be achieved. A major challenge has been the difficulty of testing and calibrating sensors at the pre-clinical stage. Little is known about fetal movement features, and clinical trials involving pregnant women can be expensive and ethically stringent. To address these issues, we introduce a novel FM simulator, which can be used to test responses of sensor arrays in a laboratory environment. The design uses a silicon-based membrane with material properties similar to that of a gravid abdomen to mimic the vibrations due to fetal kicks. The simulator incorporates mechanisms to pre-stretch the membrane and to produce kicks similar to that of a fetus. As a case study, we present results from a comparative study of an acoustic sensor, an accelerometer, and a piezoelectric diaphragm as candidate vibration sensors for a wearable FM monitor. We find that the acoustic sensor and the piezoelectric diaphragm are better equipped than the accelerometer to determine durations, intensities, and locations of kicks, as they have a significantly greater response to changes in these conditions than the accelerometer. Additionally, we demonstrate that the acoustic sensor and the piezoelectric diaphragm can detect weaker fetal movements (threshold wall displacements are less than 0.5 mm) compared to the accelerometer (threshold wall displacement is 1.5 mm) with a trade-off of higher power signal artefacts. Finally, we find that the piezoelectric diaphragm produces better signal-to-noise ratios compared to the other two sensors in most of the cases, making it a promising new candidate sensor for wearable FM monitors. We believe that the FM simulator represents a key development towards enabling the eventual translation of wearable FM monitoring garments.

## 1. Introduction

Monitoring of fetal movements (FM) has long been a subject of interest to medical and research communities due to the association between reduced FM and several fetal health conditions, such as fetal distress, placental dysfunction, fetal growth restriction, and hypoxia [1,2,3,4,5,6]. A reduction in FM often provides an early warning of fetal health complications [7,8,9,10]. For example, in a study on 305 pregnant volunteers with reduced FM after 28 weeks of gestation, 22.1% reported poor outcomes at birth, such as preterm or small-for-gestational-age births [9]. Another study on 161 singleton pregnancies resulting in stillbirths at Nottingham City Hospital from 1991 to 1997 stated that reductions in FM were reported in 54.7% of the cases [10]. A recent study on 409,175 pregnancies in the UK and Ireland found that the awareness of reduced FM through maternal sensation does not significantly reduce the risk of stillbirth [11], but this does not negate the value and impact of a method for objectively quantifying FM.

The oldest and most commonly used method of quantifying FM is through maternal sensation. Despite being a very common screening method followed by a large portion of pregnant women around the world, this method has largely been set aside by medical professionals [12]. Studies have shown that this method is highly patient dependent, especially with subtle or gentle movements [13]. Results from recent studies propose that only around 40% of the movements observed in ultrasonography are detected by maternal sensation [14,15]. While pregnant women are always counseled to report any perceived decrease in fetal movements, formal “kick counting” is actively discouraged in many contemporary guidelines for antenatal care [16,17]. Additionally, there is a lack of clear guidance on a “safe” number of perceived movements [12]. Other common methods of quantifying fetal activity include ultrasonography [18,19], MRI scanning [20], and cardiotocography [21]. Such methods need expert operators and can only be performed for short periods in clinical environments. Additionally, ultrasonography (and especially MRI) may not be available in the majority of the rural areas in low-income countries. Current clinical methods are, therefore, unsuitable for regular monitoring of fetuses and have failed to determine a reliable quantitative guideline for a normal or safe level or frequency of FM.

Building upon advances in electronics and sensor technology, several attempts to develop a low-cost, wearable, non-transmitting system for detecting FM have been reported in recent years. Most of these attempts have used accelerometer-based systems to detect low-frequency vibrations of maternal abdomens created by fetal movements. For example, Thomas et al. [22] used a single accelerometer to record FM from 27 pregnant women and achieved average true and false detection rates of 62% and 40%, respectively, compared to concurrent ultrasound scans. Using two custom-made capacitive acceleration sensors, Roy et al. [23] found 79% positive agreement with concurrent ultrasound recordings from 14 pregnant women for gross movement of fetal trunks. However, their positive detection rates for the isolated limb and breathing movements were only 36% and 21%, respectively. Mesbah et al. [24] presented a fetal activity monitor that uses three accelerometers to detect fetal activities and an additional accelerometer, placed on the mother’s chest, to detect artefacts from maternal activities. Considering the data recorded by one of the accelerometers only, they obtained positive agreements of 50%, 52%, and 76% with concurrent ultrasound detections for three different recordings. A complete analysis of the data obtained from all four accelerometers was presented by Boashash et al. [15], where special time-frequency based techniques were used to improve the detection rate by removing signal artefacts. Analyzing the data from six pregnant women, they reported overall true detection rates of 78% and 72% for two different methods; the corresponding false detection rates were 17% and 15%, respectively. By experimenting with six accelerometers, Altini et al. [25] reported that the addition of accelerometers after two does not improve the detection accuracy significantly, while the addition of a reference accelerometer to remove artefacts due to maternal activity consistently improves the detection accuracy for any number and arrangement of sensors. In a recent work from our group, Lai et al. [26] presented a new design of FM monitor based on a combination of six acoustic sensors and an inertial measurement unit (IMU). The IMU mainly consisted of an accelerometer, which was used to detect maternal body movements. The system was validated against concurrent ultrasound tests on a cohort of 44 pregnant women. It achieved a promising true positive detection rate of 78% in detecting startle movements (vigorous, whole-body movements) but performed poorly in the cases of general and breathing movements (true positive detection rates were 53% and 41%, respectively). Additionally, the average ratio of sensor-detected movements to ultrasound-detected movements was 2.4, which demonstrates a high number of false-positive detections by this system.

Although extensive efforts have been made to design FM monitors using accelerometers, the desired accuracy of detection has not been achieved and translation remains negligible. Our recent study indicates that while acoustic sensing is promising, the false-positive detection remains unacceptably high [26]. Therefore, it is important now to quantify the limitations of these sensors, look for new candidate sensors, and consider a combination of different types of sensors to further improve the accuracy of detection. This is very challenging to achieve through clinical studies on pregnant women. The “ground truth” for FM can only be provided (partially) with ultrasound testing, and repeated testing of multiple sensors against ultrasound is not feasible ethically or logistically in human studies. We, therefore, approach this problem by enabling extensive testing of sensors and their combinations, prior to a clinical testing phase, through the development of an FM simulator. The only effort towards the development of an FM simulator to date has been reported by Sazali et al. [27], who proposed a very simple design consisting of a servo-controlled kicking mechanism and a flat rubber sheet as a maternal abdomen model. However, to be able to test sensors for an FM monitor, the vibration characteristics of a maternal abdomen due to fetal kicks need to be replicated by the simulator.

In the present study, we design and fabricate a novel FM simulator considering the geometrical and material properties of a maternal abdomen to imitate its vibration characteristics. The design also includes a stretching mechanism to simulate the pre-stress present in a gravid uterus and a kicking mechanism to replicate fetal kicks in terms of the speed, duration, and reaction force from the abdomen wall. To run the simulator, we develop a software application that allows the design of simulated kicks using wide-ranging features. Finally, as a case study, we investigate performances of three vibration sensors, namely an accelerometer, an acoustic sensor, and a piezoelectric diaphragm, analyzing their relative performances when used on the simulator under various input conditions.

## 2. Design of the Fetal Movement Simulator

**Figure 1:**
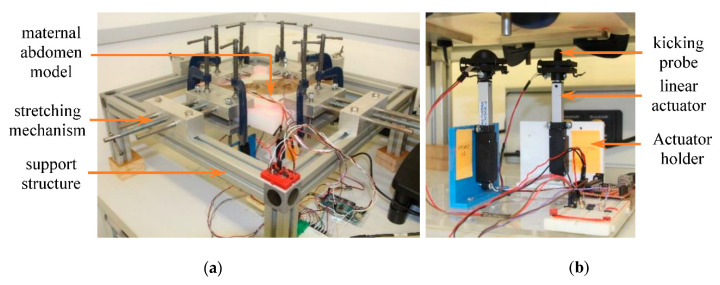
Fully assembled fetal movement (FM) simulator: (**a**) maternal abdomen model, and support structure and stretching mechanism, (**b**) kicking mechanism beneath the abdomen membrane.

The mechanical design of the FM simulator consists of three main components—maternal abdomen model, support structure and stretching mechanism, and kicking mechanism (Figure 1). Detailed descriptions of these components along with the data acquisition and software systems used in the simulator are provided in this section.

### 2.1. Design of the Maternal Abdomen Model

**Figure 2:**
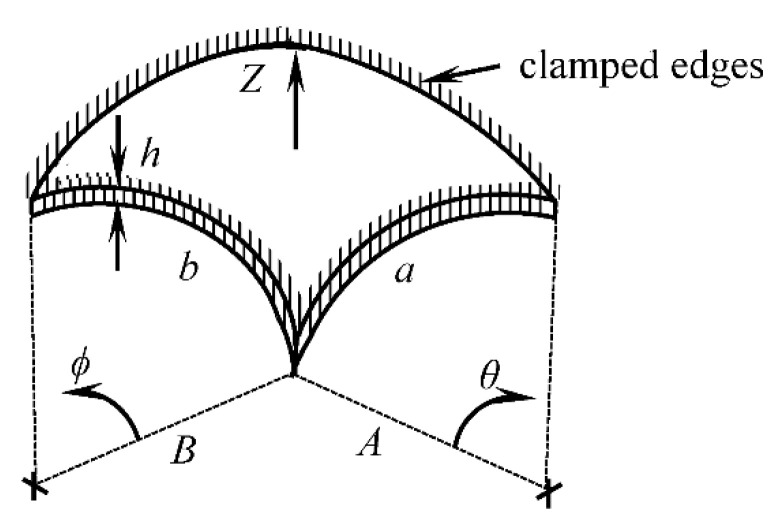
Geometry and boundary conditions of the curved single-layer membrane model of a maternal abdomen.

A single layer membrane with clamped edges was assumed to model the vibrations of the gravid abdomen due to fetal kicks. To understand which material and geometrical properties influence vibration characteristics, a mathematical model of the membrane vibration was studied. The approximate analytical formula for the natural frequency of vibration of a curved rectangular membrane with clamped edges (Figure 2), as derived by P. J. Palmer [28] using the Rayleigh’s method, is given as follows:

(1) $$f_{n}\;=\;\frac{1}{2\pi }\sqrt{\frac{D}{\rho h}\left\{72\left(\frac{7}{a^{4}}\;+\;\frac{7}{b^{4}}\;+\;\frac{4}{a^{2}b^{2}}\right)\;+\;\frac{12}{h^{2}}\left(\frac{1}{A^{2}}\;+\;\frac{1}{B^{2}}\;+\;\frac{2\mu }{AB}\right)\right\}},$$

where
*f_n_* = natural frequency of vibration (Hz) $D\;=\;\frac{Eh^{3}}{12\left(1-\mu^{2}\right)}=$ flexure rigidity (Pa·m^3^)*A*, *B* = radii of curvature (m) *E* = modulus of elasticity (Pa)*a*, *b* = length of sides (m) *µ* = Poisson’s ratio*h* = membrane thickness (m) *ρ* = density (kg/m^3^) 

The region between the fetus and the exterior surface of the maternal abdomen consists of three layers, namely fetal membrane, uterine wall, and abdominal wall. Table 1 lists the previously reported material and geometrical property values of these layers relevant to the formula for the natural frequency of vibration of the membrane (Equation (1)).

In the cases where explicit mentions of the range of property values were not found, the minimum and maximum values of the properties presented in Table 1 were calculated using “mean ± 2 × standard deviation” formula. The properties of the fetal membrane were obtained from the literature by considering it as a single layer of chorioamniotic material, whose outer surface is attached to the uterine wall. The intrauterine radius was calculated using the previously reported values of intrauterine volume [34] and assuming the spherical shape of the uterus [35]. The thickness of the abdominal wall was calculated by adding the thicknesses of the abdominal muscle and fat tissues [36]. The density of the abdominal muscle was taken from the values reported by Rachel et al. [38]. Using computer tomography images of the abdomen of nearly 2000 subjects, they obtained the density values in the Hounsfield unit (HU), which represents the attenuation of X-ray by the material and is proportional to the physical density of the material. To convert the HU value into Kg/m^3^, the linear fitting of the experimental data of the density (Kg/m^3^) and the corresponding HU value for soft human tissues presented by Uwe et al. [37] were used. Finally, the weighted average of the abdominal muscle and fat densities [37] with respect to the corresponding mean thicknesses [36] was considered as the abdominal wall density.

Weighted average values of the material properties with respect to the mean thicknesses of the corresponding layers were considered to model the single-layer maternal abdomen. Since the density of the fetal membrane was not found in the literature, only uterine and abdominal walls were considered to calculate the weighted average density of the single-layer abdomen model. For simplicity of modeling, equal radii of curvature and equal lengths of sides in both directions were considered. The inner radius of the fetal membrane was calculated by deducting its thickness from the intrauterine radius. The mean radius of curvature of the abdomen was calculated to be 118.1 mm by adding half of the overall thickness of the fetal membrane, uterine wall, and abdominal wall with the inner radius of the fetal membrane. Finally, the lengths of the sides of the membrane were considered as equal to the circumference of a semicircle with a radius equal to the radius of the abdomen. With the mean values of the above mentioned geometrical and material properties, the natural frequency of undamped free vibration of the single-layer curved model of a maternal abdomen was calculated as 33.46 Hz using Equation (1).

**Figure 3:**
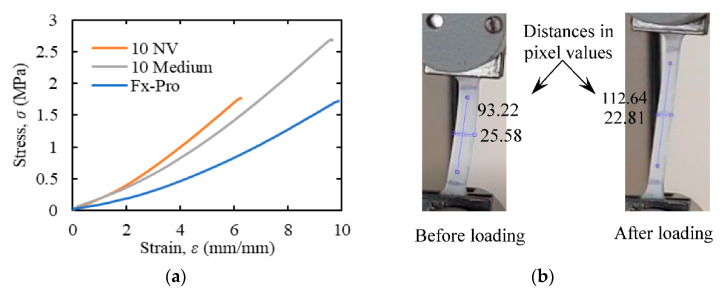
Uniaxial tensile test results for the silicon materials: (**a**) stress-strain diagram, (**b**) pixel values of the longitudinal and lateral dimensions of a specimen before and after applying a tensile load to calculate the Poisson’s ratio of the material. ASTM D412 standard was followed to perform the tests.

After a preliminary comparison of relevant characteristics of some suitable candidate materials (e.g., silicone, thermoplastic elastomer, alginate gel, etc.) for modeling the maternal abdomen, silicone was selected due to its durability, ease of manufacturing, and availability of variants with required material properties. The uniaxial tensile test was performed on three variants of silicone, namely Dragon skin FX-pro, Dragon Skin 10 NV, and Dragon Skin 10 medium, obtained from the Smooth-on, Inc. (Macungie, PA, USA), following the ASTM D412 standard. The modulus of elasticity was determined from the stress vs. strain curve at 100% elongation (Figure 3a), and the Poisson’s ratio was determined by analyzing images of the specimens before and after the elongation (Figure 3b).

The uniaxial tensile test was performed on three samples for each of the silicone variants, and the median value was considered as the average property value for that variant (Table 2). Based on the material properties of different variants of silicone (Table 2) and the property requirements for the maternal abdomen model (Table 1), Dragon Skin 10 NV was selected for modeling the abdomen.

**Figure 4:**
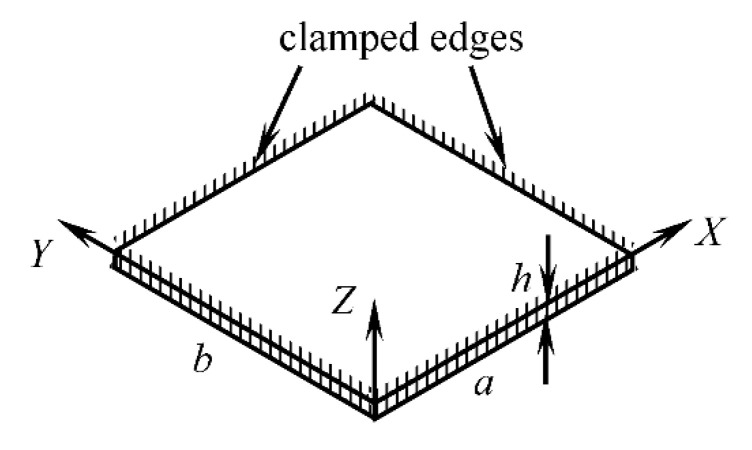
Geometry and boundary conditions of the single-layer flat rectangular model of a maternal abdomen.

For design simplification of the support structure and stretching mechanism, a flat rectangular membrane (Figure 4) with the natural frequency of vibration equal to the natural frequency of vibration of the curved abdomen model (i.e., 33.46 Hz) was used in the simulator. For a flat rectangular membrane with clamped edges, the approximate analytical solution for the natural frequency of free damped vibration, as derived by Milomir M. S. by using the Galerkin’s method [39], is given as

(2) $$f_{nd}\;=\;\frac{1}{2\pi }\sqrt{\frac{\wedge D}{\rho ha^{3}b}\;-\;{\left(\frac{k}{2\rho h}\right)}^{2}},$$

where
*f_nd_* = natural frequency of free damped vibration (Hz)*∧* = 36.11^2^ (square membrane, 1st mode of vibration)*k* = coefficient of damping (N·s/m).

Neglecting the effect of damping (*k* = 0), the natural frequency of undamped free vibration of the flat membrane can be obtained from Equation (2) as

(3) $$f_{n}\;=\;\frac{1}{2\pi }\sqrt{\frac{\wedge D}{\rho ha^{3}b}}.$$

For the selected silicone material (Dragon Skin 10 NV), the length of the sides of a square membrane with the natural frequency of vibration equal to the natural frequency of the curved abdomen model (= 33.46 Hz) was calculated to be 171 mm using Equation (3).

Expressions for the damping ratio (*ζ*) and the time constant (*τ*) were also derived from the solution of Milomir M. S. [39] to investigate the transient characteristics of the membrane vibration, which are given as follows

(4) $$\zeta \;=\;\sqrt{1\;-\;\frac{\rho ha^{3}b}{\wedge D}{\left(2\pi f_{nd}\right)}^{2}},$$

(5) $$\tau \;=\;\frac{2\rho h}{k}.$$

### 2.2. Design of the Support Structure and Stretching Mechanism

**Figure 5:**
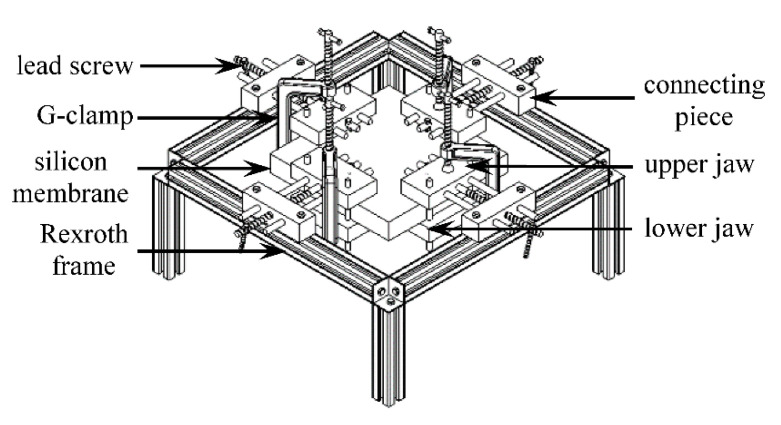
Fully assembled CAD design of the support structure and stretching mechanism.

The support structure consists of four clamping mechanisms to hold the silicone membrane from four sides and an aluminum Rexroth frame to support the clamps and provide the required elevation for the kicking mechanism to fit beneath the membrane (Figure 5). To assemble the frame, three-way connectors with self-tapping screws were used at each of the four corners of the frame. Each clamp is attached to the frame using two T-nuts to allow for the clamp attachment piece to slide along the strut and provide added flexibility in the positioning of the clamp. The clamp components are made of aluminum bars and are connected through mild steel rods. The upper jaw of the clamp is connected to the attachment piece through a 10 mm lead screw at the middle and two 10 mm non-threaded stabilizing rods at two sides. G-clamps were used to secure the upper and lower jaws tightly after connecting them through two 8 mm non-threaded vertical rods.

To simulate the strained condition of a gravid abdomen, the membrane was stretched by rotating the lead screw after clamping. The tensile load was assumed to be uniform throughout the membrane and was calculated from the elongation of the membrane. For this purpose, the relation between the elongation and the tensile stress was derived. Considering isotropic material property and obeyance to Hooke’s law, the relationship between stresses and strains for biaxial loading can be given as

(6a) $$\epsilon_{x}\;=\;\frac{1}{E}\left(\sigma_{x}\;-\;\mu \sigma_{y}\right),$$

(6b) $$\epsilon_{y}\;=\;\frac{1}{E}\left(\sigma_{y}\;-\;\mu \sigma_{x}\right),$$

where
$\epsilon_{x},\epsilon_{y\;}=$ normal strains along *X* and *Y* axes, respectively$\sigma_{x},\sigma_{y}=$ normal stresses along *X* and *Y* axes, respectively (Pa).

For a square membrane with the length of side *a* and equal tensile stress (*σ*) along both axes, the formula for the elongation (*δ*) can be derived from Equations (6a) & (6b) as

(7) $$\delta =\frac{a\sigma }{E}\left(1-\mu \right).$$

Uterine wall tensions for different gestational ages (obtained from the literature review) and the elongations of the membrane required to produce the corresponding tensions, which were calculated by using Equation (7) and the material properties of the membrane (Table 2), are listed in Table 3. Applying these elongations, the stretched condition of the maternal abdomen for different gestational ages can be created in the FM simulator.

### 2.3. Design of the Kicking Mechanism

**Figure 6:**
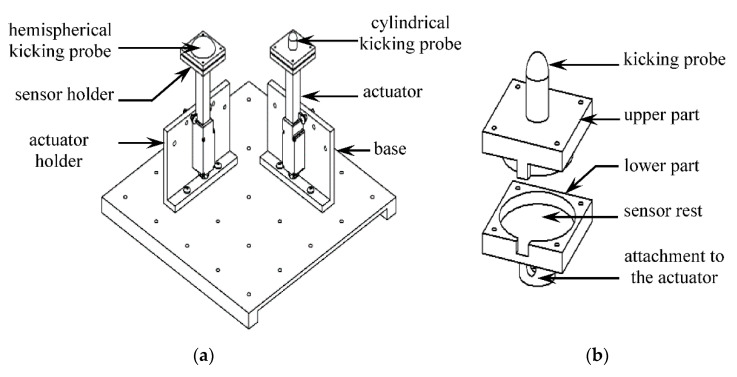
CAD design of the (**a**) fully assembled kicking mechanism, (**b**) sensor holder.

The kicking mechanism consists of two sets of actuators, sensor holders, kicking probes, and actuator holders along with a base to support the whole mechanism (Figure 6a). Actuonix (Victoria, BC, Canada) L16-R linear servo actuator (Model No.: L16-50-63-6-R) was used to simulate the fetal kicks in the simulator. The position of the servo was controlled by 5V pulse-width modulated (PWM) signals from an Arduino Mega board (from Arduino, Torino, Italy). The whole process was automated via MATLAB’s “writePosition ()” function, which takes the position of the actuator as a position variable that varies between 0 (fully retracted) and 1 (fully extended). To accurately control the position, the actuators were calibrated by measuring their stroke length for different values of the position variable. When connected to a 5V dc supply, the maximum stroke length and the no-load speed of the actuators were found to be 146 mm and 12.43 mm/s, respectively.

The actuator holder is a 3D-printed structure to hold the actuator in proper position and orientation beneath the silicone membrane (Figure 6a). The holes in the vertical and horizontal planes of the actuator holder serve as the points of attachment for the actuator and the base, respectively. The actuator can be positioned along three different angular orientations in a holder relative to the horizontal plane, namely 90° (perpendicular), 60°, and 55°, to generate kicks with different angles of impact. The base of the kicking mechanism is made of a 250 mm × 250 mm × 5 mm acrylic sheet with 4 mm diameter holes distributed along the whole horizontal plane to ensure multiple arrangements of kicking positions when both the actuators run simultaneously.

The sensor holder consists of a two-part sandwich-like structure that encloses a force sensor in the middle (Figure 6b) to measure the reaction force due to kicking. Piezoresistive force sensor FlexiForce A401 from Tekscan Inc. (South Boston, MA, USA, 2019) was selected to be used for this purpose due to its suitability of dimension (25.4 mm diameter), good repeatability (< ±2.5%), and appropriate range of measurement (0–111 N) [40]. Two force sensors were used with two actuators, and their calibrations were performed using a set of known calibration weights.

The kicking probe is located on the top of the sensor holder and is 3D printed as an integrated structure with the sensor holder (Figure 6b). A 10 mm diameter cylindrical probe was used to represent the geometry of a 20-week old fetal foot [31], and a 30 mm diameter hemispherical probe was used to characterizes the geometry of an older (30 weeks) fetal foot.

### 2.4. Data Acquisition System

**Figure 7:**
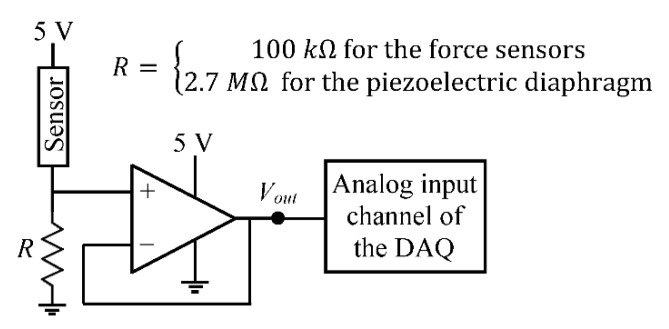
Diagram of the circuit used between the sensor output and the DAQ input in the cases of the force sensors and the piezoelectric diaphragm. LM 358 op-amp was used to design the voltage follower circuit.

USB 6212, a multifunction I/O device from National Instruments (Austin, TX, USA), was used as the data acquisition (DAQ) system for the FM simulator. It has 16 analog input channels, which can sample data at a maximum rate of 400 kHz for a single channel with a resolution of 16 bit. The output pins of the force sensors were connected to a voltage divider circuit followed by a voltage follower circuit (LM 358 op-amp), and the output from the voltage follower was connected to the analog input channel of the DAQ system as shown in Figure 7. A sampling rate of 2 kHz was used for recording the data during the experiments.

The recorded data from the force sensors were first passed through a low pass filter (4th order zero-phase Butterworth filter, 50 Hz cut-off frequency) and then converted into force values based on the calibration results of the corresponding force sensor. Additionally, the offset error in the sensor output due to the initial compression of the sensor in the sensor holder was subtracted from the readings.

### 2.5. Software for the Simulator

**Figure 8:**
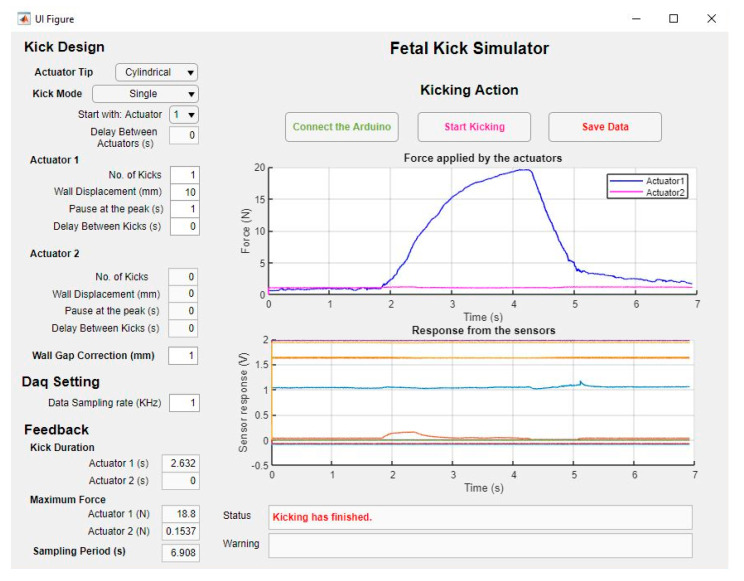
The graphical user interface of the software application developed to run the FM simulator.

A MATLAB (MathWorks, Natick, MA, USA) -based software application (Fetal Kick Simulator) was developed to run the simulator and record data from the sensors (Figure 8). The application controls the actuators by sending PWM signals from an Arduino Mega board based on a user-defined kick profile. Several parameters, such as the number of kicks, desired wall displacement, pause at the peak, etc., are inputted by the user. Additionally, users can select one of four defined kick modes from a drop-down list in the graphical user interface (GUI) of the software to specify the sequence of movements of the actuators (Table 4).

The sensor data are recorded by the DAQ system in the background and are sent to the computer via serial communication in every 0.25 s. The software updates the data and plots them instantaneously to display almost real-time changes in sensor outputs. At the end of the recording, the user can save the data before starting the next recording. A video recording of the hardware and the software systems running simultaneously is added as Supplementary Material with the paper.

## 3. Characteristics of the Simulator

After holding the silicone membrane with the gripping mechanism, the area of the flat abdomen model was found as 167 × 167 mm. For simulating the stretched condition of a 30 weeks pregnant abdomen, a bi-axial loading of 22.8 kPa was applied to the membrane through elongations of 11 mm along both axes of the membrane, which was calculated using the formula mentioned in Table 3. The thickness of the membrane was measured as 39 mm. With these geometrical properties and the material properties mentioned in Table 2, the natural frequency of undamped free vibration of the silicone membrane was calculated to be 31.24 Hz (by using Equation (3)), which is very close to the calculated frequency of vibration of the curved abdomen model (33.46 Hz). However, because of the effect of damping, the natural frequency of free vibration of the membrane, as measured by the acoustic sensor and the accelerometer, was found to be 18.7 Hz (Table 5), which is much lower than the calculated natural frequency.

To understand the transient characteristics of the membrane vibration, the damping ratio was calculated as 0.80 using Equation (4), and the time constant was calculated as 6.4 ms using Equation (5). This value of the damping ratio signifies that the vibration of the abdomen membrane is an underdamped case (as *ζ* < 1), and it will decay rapidly (as *ζ* is close to 1). The time constant of 6.4 ms signifies that in the case of free damped vibration, the amplitude will decay by more than 99% of its initial value within a period of 32 ms (= 5*τ*).

Table 6 shows the comparison of the characteristics of real fetal kicks as reported by Verbruggen et al. [30] with the simulated kicks obtained from the simulator with the cylindrical and the hemispherical kicking probes. For the simulated kicks, the durations presented in this table are the minimum kick durations obtained by running the actuators at their maximum speeds (12.43 mm/s at the no-load condition), which can be easily increased by slowing down the actuators. As the reaction force from the membrane depends on the location of the kick impact point relative to the clamped edges, 40 measurements were taken at different locations of the membrane to calculate the average kick reaction force for each value of the wall displacement. These results demonstrate that for the higher value of the wall displacement (close to the upper limit of the range), the cylindrical kicking probe produces reaction forces similar to 20-week old fetuses, and the hemispherical kicking probe produces reaction forces similar to 30-week old fetuses. However, for the lower value of the wall displacement (close to the lower limit of the range), the hemispherical kicking probe produces reaction forces more similar to 20-week old fetuses.

To verify the reliability of the current FM simulator in producing repeatable and consistent simulations of abdominal vibrations due to fetal kicks, multiple measurements were used to obtain the simulator characteristics as reported in Table 5 and Table 6. Table 5 shows that the measurements of the natural frequency of vibration of the simulator abdomen model obtained independently from two different sensors (accelerometer and acoustic sensor) are very close to each other (18.70 Hz for the accelerometer and 18.75 Hz for the acoustic sensor), and the standard deviations (SD) (obtained from 10 measurements) are very small (0.10 for the accelerometer and 0.17 for the acoustic sensor). Similarly, the standard deviations of the kick reaction forces (obtained from 40 sets of measurements) are also quite reasonable (ranges from 1.66 to 3.92 N) considering the mean value of the forces (ranges from 14.84 to 44.2 N) and the variation of the membrane resistance due to the location of the kick impact point (Table 6). These results show that the performance of the simulator is consistent and repeatable under the experimental conditions reported in this paper.

## 4. Testing of the Sensors on the Simulator

**Figure 9:**
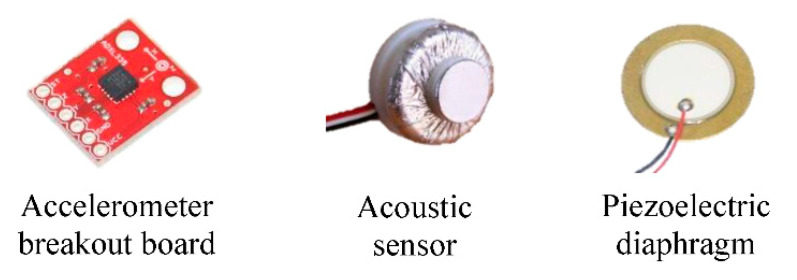
Vibration sensors that were tested on the FM simulator as a case study in this research. The detailed specification of the accelerometer breakout board and the piezoelectric diaphragm can be found in [41,42], respectively. The design of the acoustic sensor is adopted from [43].

The developed FM simulator can be used to test any kind of candidate sensors for FM monitors to evaluate their ability to detect abdominal vibrations due to fetal kicks. In this study, three types of passive vibration sensors, namely an accelerometer, an acoustic sensor, and a piezoelectric diaphragm (Figure 9), were tested, and the outputs from these sensors were stored for post-processing. A sampling rate of 2 kHz was used for recording the data during the experiments.

### 4.1. Selection of the Sensors

The breakout board for ADXL335 from SparkFun Electronics (Niwot, CO, USA, 2014) was selected as a suitable accelerometer for testing. ADXL335 is a 3-axis MEMS accelerometer from Analog Devices Inc. (Norwood, MA, USA, 2009), which has a range and sensitivity of ±3 g and 300 mV/g, respectively, with extremely low noise and power consumption rate [41]. The breakout board includes 0.1 µF capacitors connected to the output pins of the sensor axes to limit their bandwidth to 50 Hz [41].

The acoustic sensor tested on the FM simulator is similar to the sensors used by Lai et al. [26] in their wearable FM monitor. The design of this sensor was originally adapted from the design first presented by Posatskiy and Chau [43]. It consists of a diaphragm covering a sealed chamber and a microphone (SPU1410LR5H-QB, Knowles Acoustics, Itasca, IL, USA, 2012). The chamber is a 3D printed structure, which is dimensioned to capture low-frequency vibrations. The microphone is positioned at the opposite end of the chamber to record the pressure changes when the diaphragm is disturbed. A frequency sweep using a custom-made vibration rig has shown that the sensor can detect low-frequency vibrations ranging from 1 Hz to 100 Hz with the peak response lying below 50 Hz [44].

A 20 mm diameter piezoelectric diaphragm from Murata Electronics (manufacturer part no. 7BB-20-6L0, Kyoto, Japan, 2009) was selected as the third type of vibration sensor to be tested on the FM simulator. This sensor consists of a 14 mm diameter piezoelectric ceramic plate glued to a 20 mm diameter brass plate [42]. Two lead wires are soldered to the plates, which are used to measure the voltage difference between them. Similar to the force sensors used in the kicking mechanism, the output from the piezoelectric diaphragm was connected to the analog input channel of the DAQ system through a voltage divider and a voltage follower circuit (LM 358 op-amp), as shown in Figure 7. Output ports of the rest of the sensors were directly connected to the analog input channels of the DAQ system.

### 4.2. Processing of Sensor Data

Recorded data from the sensors were first passed through a bandpass filter (4th order zero-phase Butterworth filter) with a suitable passband frequency of 1–40 Hz, which was selected based on the expected frequency of vibration of the membrane due to kicking as calculated in Section 2.1. In the case of the accelerometer, further processing of data was done on the magnitude (Euclidian norm) of the acceleration, *A*, which was calculated as

(8) $$A\;=\;\sqrt{A_{x}^{2}\;+\;A_{y}^{2}\;+\;A_{z}^{2}},$$

where *A_x_*, *A_y,_* and *A_z_* represent the components of acceleration along the *X*, *Y,* and *Z* axes, respectively.

**Figure 10:**
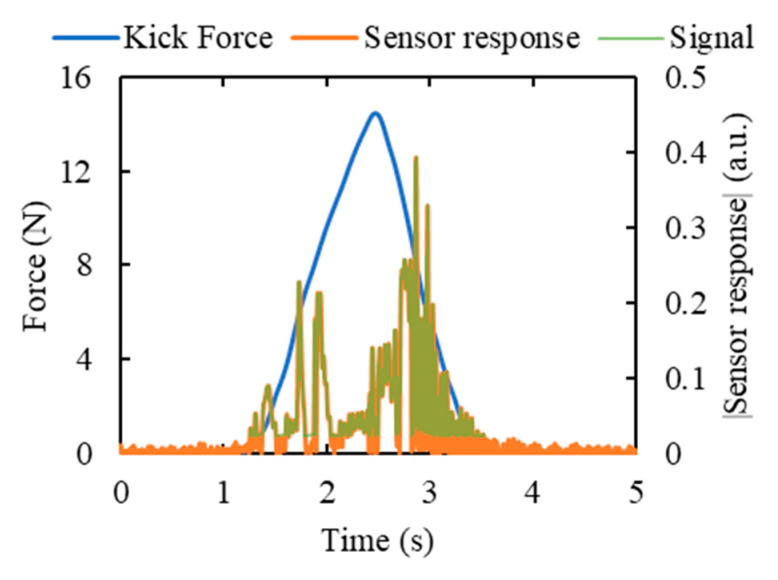
Determination of the part of the sensor response that is considered as the signal due to kicking based on the condition mentioned in Equation (10). Response from the acoustic sensor due to a simulated kick of 10 mm wall displacement using the cylindrical kicking probe is used in this figure. The distance between the sensor and the kick impact point was 5 cm.

Matrices that were used to compare the performance of different sensors are sensor response, signal energy, signal-to-noise ratio (SNR), and the dominant frequency mode in the output signal. The following steps were performed to calculate the signal energy and the SNR:

1. A noise estimate, *e*, was determined by taking the average of the absolute value of sensor response (*V_i_*) for a window of time *w* (=2 s) during a period when no kick action was going on.
  (9) $$e\;=\;\frac{1}{F_{s}w}\sum_{i=1}^{F_{s}w}\left|V_{i}\right|,$$
  where *F_s_* is the frequency of data sampling. The time window was taken at a period sufficiently away (= 6 s) from the previous kick action to ensure the complete decay of oscillation due to the previous kick.
2. The signal due to kick, *S*, was defined as the part of sensor response that exceeds the threshold level *h* = *me*, where *m* is a multiplier evaluated from trial and error:
  (10) S={x∈|V| |x > h}.
  *m* = 5 was found to generate the most suitable threshold value for the majority of the cases, and this value was, therefore, used for all the datasets. Figure 10 demonstrates the application of this algorithm in the case of acoustic sensor response due to a simulated kick.
3. The rest of the data were considered as noise *N*:
  (11) N={x∈V∣x ≤ h}.
4. Energy, *E*, of the signal and the noise were calculated as
  (12) $$E_{W}=\sum_{x\in W}{\left|x_{i}\right|}^{2},$$
  where *W* = *S* for signal and *W* = *N* for noise.
5. Finally, the SNR was calculated as
  (13) $$SNR=10\log_{10}\left(\frac{E_{S}/n_{S}-E_{N}/n_{N}}{E_{N}/n_{N}}\right)\;\;\left(dB\right),$$
  where *n_S_* and *n_N_* are the number of elements in the signal and the noise data, respectively. The noise power was assumed to be constant throughout the whole sensor response. It was, therefore, subtracted from the overall signal power to determine the power due to the kick action alone.

## 5. Results and Discussion

This section describes the results obtained from the performance testing of three vibration sensors, namely an accelerometer, an acoustic sensor, and a piezoelectric diaphragm, using the FM simulator. All the sensors were attached tightly to the exterior surface of the simulator membrane using adhesive tapes, and the center of the membrane was maintained as the middle point between the sensor and the kick impact point. All kicks were produced at the maximum actuator speed.

### 5.1. Comparative Responses from Different Sensors

**Figure 11:**
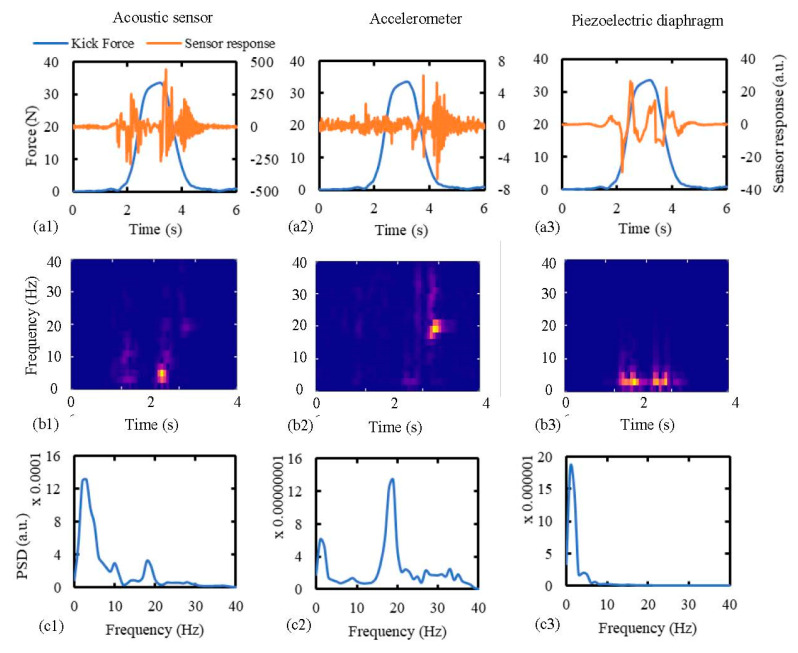
Comparison of signal characteristics of the sensors due to simulated fetal kicks in terms of: (**a1,a2,a3**) sensor response, (**b1,b2,b3**) spectrogram, and (**c1,c2,c3**) power spectral density (PSD) distribution. The abdomen model was subjected to simulated kicks of a 30-week fetus and the sensors were attached to a distance of 5 cm from the kick impact point. Datasets of 10 repeated kicks were used to generate the PSD distributions.

To study the comparative responses, all the sensors were placed at a distance of 5 cm from the kick impact point. Simulated kicks of 14 mm wall displacement were applied using the hemispherical probe to replicate kick characteristics of a 30-week old fetus (Table 6). Kicks of a 30-week old fetus were chosen here, because this time point provided the best match between actual and simulated fetal kicks, as shown in Table 6. Sensor performances are displayed in Figure 11 in terms of sensor response, spectrogram, and power spectral density (PSD) distribution. Hann window of length 0.5 s and 80% overlap between the segments were used to obtain the spectrograms, which resulted in frequency and time resolutions of 2 Hz and 100 ms, respectively. The PSD distributions were obtained using the Welch method and a dataset of 10 similar kicks. Hann window of length 1s and 50% overlap between segments were used to generate the PSD distributions.

Figure 11a shows that the acoustic sensor and the piezoelectric diaphragm were responsive to the kick during the majority of the kick duration. Though the accelerometer detected the starting of the kick, it was mainly responsive near the end of the return stroke, which is the period when the membrane starts vibrating freely. This demonstrates the accelerometer’s inability to detect membrane vibrations during the period of forced movements by the kicking probe. This can also be observed from the spectrograms of the sensor responses (Figure 11b), which show that after the 4th second (near the end of kick) the membrane was vibrating at around 19 Hz, which is equal to the natural frequency of vibration of the membrane. Both the acoustic sensor and the accelerometer picked up this vibration, but the piezoelectric diaphragm did not show any response. This characteristic of the sensors is further observed in the PSD distribution curves (Figure 11c), which show that the power of the piezoelectric signal was completely concentrated below 10 Hz. In the cases of the acoustic sensor and the accelerometer, concentrations of signal power around 2 Hz and 19 Hz were observed. However, the main peak occurred around 2 Hz in the case of the acoustic sensor and around 19 Hz in the case of the accelerometer. This PSD distribution of the acoustic signal also conforms with the PSD distribution obtained from the acoustic signals due to the real fetal kicks, as reported by Lai et al. [26].

### 5.2. Effect of Wall Displacement and Kick Distance on the Sensor Response

**Figure 12:**
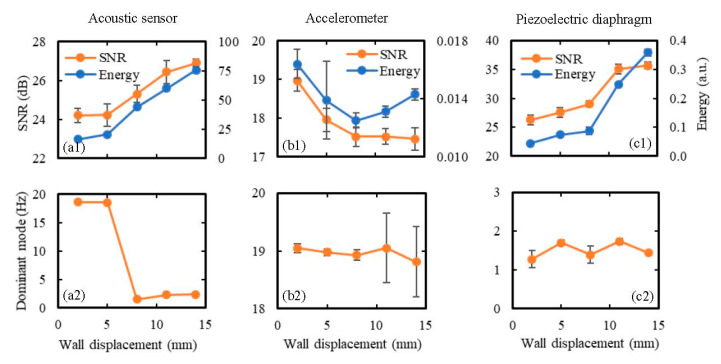
Effect of wall displacement on the sensor performance in terms of: (**a1,b1,c1**) SNR and signal energy, and (**a2,b2,c2**) the dominant frequency mode. The sensors were attached to the model abdomen membrane at 5 cm away from the kick impact point, and the wall displacement due to kicking was varied from 2 to 14 mm using the hemispherical kicking probe. Ten sets of data were taken for each wall displacement and the results are shown in terms of the mean values as the data points on the graph and the standard deviations as the error bars.

To study the effect of wall displacement on the sensor performance, all sensors were placed 5 cm away from the kick impact point and were subjected to kicks of wall displacements ranging from 2–14 mm using the hemispherical kicking probe. Each kick was repeated 10 times to evaluate the mean and the standard deviation of the output signal parameters, and the obtained results are shown in Figure 12 in terms of SNR, signal energy, and the dominant frequency mode. It can be seen from this figure that in the cases of the acoustic sensor and piezoelectric diaphragm, both SNR and signal energy increased with the increase in wall displacement (Figure 12(a1,c1)). For the accelerometer, SNR decreased with the increase in wall displacement, and signal energy initially decreased and then increased with the increase in wall displacement (Figure 12(b1)). Additionally, changes in these parameters (SNR and signal energy) were comparatively smaller in the case of the accelerometer compared to the other two sensors. This happened because the accelerometer is mainly responsive to the free vibration of the membrane (at around 19 Hz), which is much less affected by the change in wall displacement compared to the vibrations during the forced movement of the membrane. This is also evident from the consistent dominant mode of acceleration signal around 19 Hz (Figure 12(b2)) with the increase in wall displacement. The increase in wall displacement also did not affect the dominant mode in the case of the piezoelectric diaphragm, which maintained a value between 1–2 Hz (Figure 12(c2)). Finally, in the case of the acoustic sensor, the dominant mode changed from around 19 Hz to a lower frequency value below 3 Hz with the increase in wall displacement (Figure 12(a2)), which demonstrates the acoustic sensor’s ability to respond effectively to both the frequency ranges and the fact that the duration of the low-frequency vibration increases more with the increase in the wall displacement compared to the duration of the free vibration of the membrane.

**Figure 13:**
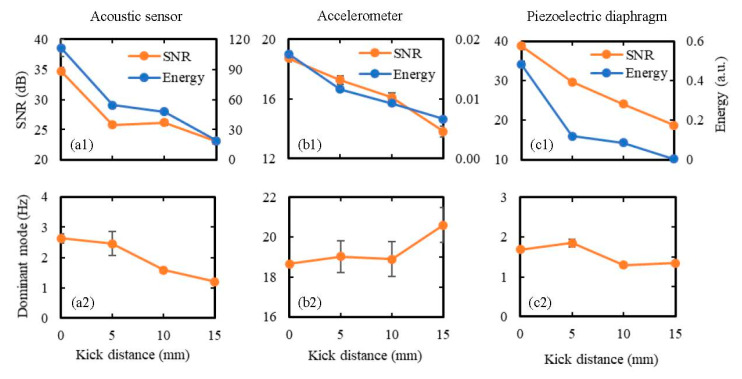
Effect of kick distance (i.e., the distance between the sensor and the kick impact point) on the sensor performance in terms of: (**a1,b1,c1**) SNR and signal energy, and (**a2,b2,c2**) the dominant frequency mode. The kick distance was varied from 0–15 cm while the abdomen was subjected to kicks of 10 mm wall displacement using the hemispherical kicking probe. Ten sets of data were taken for each kick distance, and the results are shown in terms of the mean values as the data points on the graph and the standard deviations as the error bars.

To study the effect of the distance between the sensor and the kick impact point (i.e., kick distance) on the sensor performance, the kick distance was varied from 0–15 cm, while keeping the wall displacement fixed at 10 mm. Again, the hemispherical kicking probe was used, and each kick was repeated 10 times to evaluate the mean and the standard deviation of output signal parameters. The results of the tests are shown in Figure 13 in terms of SNR, signal energy, and the dominant frequency mode. As expected, both SNR and signal energy decreased gradually with the increase in kick distance for all three sensors as the vibration of the membrane becomes weaker with the increase in the sensor’s distance from the kick impact point. There was no significant effect of the kick distance on the dominant signal mode for any of the sensors. Again, changes in SNR and signal energy were much smaller in the case of the accelerometer compared to the acoustic sensor and the piezoelectric diaphragm.

Finally, the best SNR was achieved from the piezoelectric diaphragm in most of the cases, as shown in both Figure 12 and Figure 13, meaning that the piezoelectrical diaphragm produced better signal quality compared to the other two sensors. The accelerometer signal was found to be least affected by the changes in wall displacement and kick distance, which indicates that it will be difficult to predict the location and intensity of kicks accurately using a fully accelerometer-based FM monitor.

### 5.3. The Input Thresholds for the Sensors

**Figure 14:**
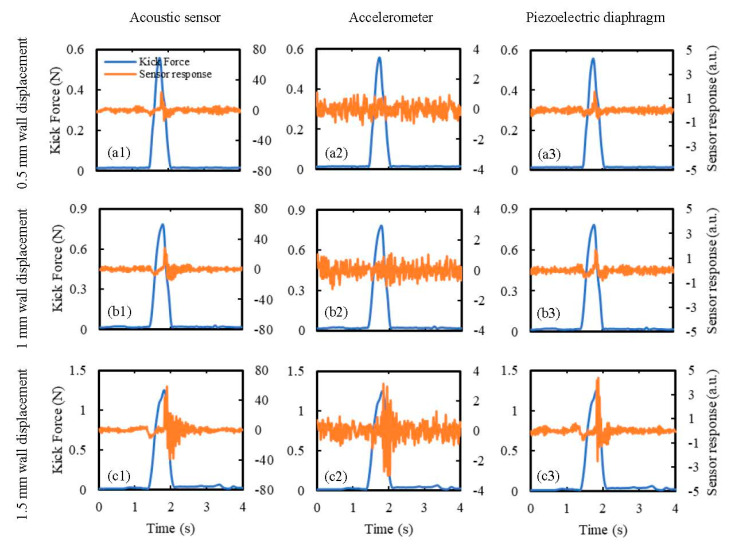
Determination of the minimum input threshold wall displacements for the sensors. Sensor responses were determined for kicks of wall displacements of: (**a1,a2,a3**) 0.5 mm, (**b1,b2,b3**) 1 mm, and (**c1,c2,c3**) 1.5 mm using the cylindrical kicking probe. The sensors were attached to a distance of 20 cm away (maximum possible for the current simulator) from the kick impact point.

The input threshold values of the sensors, i.e., the minimum input value recognized by the sensors, are expressed in terms of the minimum wall displacements due to kicking required by the sensors to recognize the kick. These values were determined by placing the sensors at the maximum possible kick distance (for the simulator) and subjecting them to the minimum possible wall displacement (for the simulator) by the weakest (in terms of the produced kick impact) available kicking probe for the current FM simulator. A length of 20 cm was the maximum possible consistent distance between the sensor and the kick impact point for the simulator to avoid attaching sensors close to the gripping mechanisms at the boundaries. Due to the 0.4 mm repeatability of the actuators [45], 0.5 mm was considered as the lowest reliable wall displacement that can be produced by the current kicking mechanism. The cylindrical kicking probe was considered as the weaker kicking probe as it produces less kick reaction force for any specific wall displacement compared to the hemispherical kicking probe (Table 6). All the sensors were, therefore, attached to a distance of 20 cm away from the kick impact point along a diagonal of the membrane and were subjected to a kick of 0.5 mm wall displacement using the cylindrical kicking probe. The obtained results are shown in Figure 14a, which shows that, while the acoustic sensor and the piezoelectric diaphragm recognized the kick clearly, the accelerometer did not have any noticeable change in its response. By gradually increasing the wall displacement by the steps of 0.5 mm, the threshold wall displacement for the accelerometer was found to be 1.5 mm (Figure 14c). The significance of this finding is that, while the smaller input threshold values of the acoustic sensor and the piezoelectric diaphragm make them more suitable for detecting weaker fetal activities compared to the accelerometer, they also make the acoustic sensor and the piezoelectric diaphragm more prone to signal artefacts compared to the accelerometer. 

The input threshold values may change based on the size of the maternal abdomen. In the case of a larger maternal abdomen, or later in gestation, two sensors on two sides of the abdomen may be needed to reduce the maximum possible distance between the sensor and the kick impact point. The results presented by Altini et al. [25] showed that using two accelerometers instead of one led to substantial improvement in the detection accuracy. In their case, any further increase in the number of sensors did not lead to any significant improvement in the detection accuracy.

### 5.4. Summary of Results

The main results can be summarized into four key findings regarding the response characteristics of the sensors when subjected to simulated fetal kicks. The first key finding is the differences between the sensors in terms of their responsiveness to different frequency ranges of membrane vibrations during the simulated kicks. While the acoustic sensor and the piezoelectric diaphragm responded strongly to low-frequency vibrations below 10 Hz, the accelerometer mainly responded to the free vibration of the membrane at around 19 Hz. This characteristic makes the acoustic sensor and the piezoelectric diaphragm better equipped to determine the kick duration compared to the accelerometer. Additionally, the acoustic sensor also responded effectively to the free vibration of the membrane. The second key finding is the differences between the sensors in terms of their responses to the changes in the wall displacement (or the reaction force) due to kicking, and the distance between the sensor and the kick impact point. The accelerometer output was found to be significantly less affected by alterations to these parameters compared to the acoustic sensor and the piezoelectric diaphragm, which makes the accelerometer relatively less capable of determining the intensity and location of a kick compared to the other two sensors. The third key finding is the differences between the sensors in terms of their minimum input threshold wall displacement values. The acoustic sensor and the piezoelectric diaphragm had a lower input threshold value compared to the accelerometer, which makes the acoustic sensor and the piezoelectric diaphragm more capable of detecting weaker fetal movements and more prone to signal artefacts compared to the accelerometer. Finally, the piezoelectric diaphragm had the best SNR for most of the input conditions, which signifies its superiority in terms of the signal quality and makes it a promising candidate sensor for fetal movement monitors. While these results establish the baseline performance of these sensors, more tests are needed to statistically compare the performance of the sensors.

## 6. Conclusions

This paper presents the most comprehensive attempt to simulate vibrations of a maternal abdomen due to fetal kicks in a laboratory set-up to date, fulfilling the unmet need for a pre-clinical facility for testing and calibration of vibration sensors for fetal movement (FM) monitors. We have designed the membrane (representing a gravid abdomen) of the simulator by incorporating relevant material and geometrical properties of gravid abdomens. We have analytically calculated the natural frequency of undamped free vibration of a curved model of gravid abdomens as 33.5 Hz and experimentally determined the actual natural frequency of an equivalent flat model of the abdomen in our simulator as 19 Hz, due to damping effects. The simulator imitates the stretched condition of a gravid abdomen by applying a biaxial tensile load on the membrane. By comparing with the available data for real fetal kicks, we have demonstrated that the designed kicking mechanism can replicate the kicks of 20 and 30-week old fetuses by using 10 mm diameter cylindrical and 30 mm diameter hemispherical kicking probes, respectively, in terms of wall displacement, kicking force, and kick duration.

As a case study, we have tested three candidate sensors for wearable FM monitors, namely an accelerometer, an acoustic sensor, and a piezoelectric diaphragm, on the simulator to investigate their performance. Analysis of the responses has revealed some novel characteristics of these sensors. For example, the acoustic sensor and the piezoelectric diaphragm were responsive to kicks during the whole kick duration because of their ability to respond to the low-frequency vibrations (<10 Hz) due to the forced movement of the membrane. Additionally, the acoustic sensor also effectively detected the free vibration of the membrane (at 19 Hz), which mainly happened at the end of the kick. However, the piezoelectric diaphragm was not responsive to the free vibration of the membrane at all. Conversely, the accelerometer was mainly responsive to the free vibration of the membrane, and therefore, although it recognized the start of the kicks, it mainly responded to inputs near the end of the kicks. These findings have also been supported by their respective spectrograms and PSD distributions. The accelerometer was significantly less responsive to the changes in wall displacement and kick distance than the other two sensors. These results indicate that it will be easier to obtain the duration, intensity, and location of the kicks from FM monitoring systems based on acoustic sensors and piezoelectric diaphragms compared to a fully accelerometer-based system. The piezoelectric diaphragm was found to produce the best SNR for most of the input conditions, which signifies its superiority in terms of the signal quality. Finally, the threshold wall displacements for the acoustic sensor and the piezoelectric diaphragm were found to be smaller compared to the acoustic sensor. This signifies that the acoustic sensor and the piezoelectric diaphragm have better abilities to detect weaker fetal activities compared to the accelerometer, but they are more prone to signal artefacts compared to the accelerometer. All these findings indicate that a combination of different types of sensing modalities will be more suitable to detect and characterize fetal activities compared to systems based on a single type of sensor.

In summary, we have created a facility for preclinical testing of sensors for FM monitors for the first time and demonstrated its capability. We have revealed novel characteristics of the responses of acoustic sensors, accelerometers, and piezoelectric diaphragm sensors to simulated fetal movements, providing valuable insights into optimally designing novel FM monitors. Future studies will involve the optimization of the number and arrangement of sensors and the study of the effect of other input conditions, such as the speed, duration, and simultaneous occurrence of kicks. Once the sensor combination is selected based on the test results from the simulator, they can be embedded in a wearable garment along with a miniaturized DAQ system and used for the clinical testing of the system. We, therefore, believe that the FM simulator presented in this study will be a major addition to the design cycle of the FM monitors.

## Figures and Tables

**Table 1:** Previously reported values of material and geometrical properties of maternal abdomens.

Layer	Material Properties, Mean (Min–Max)			Geometrical Properties, Mean (Min–Max)	
	Young’s Modulus, (kPa)	Poisson’s Ratio	Density (Kg/m^3^)	Thickness (mm)	Radius (mm)
Fetal membrane [29,30,31]	7100 (3200–13,700)	0.40	--	0.19 (0.14–0.28)	--
Uterine wall (30 week gestational age) [30,31,32,33,34,35]	586	0.40	1052	6.90 (5.0–10.50)	99 (89–117)
Abdominal wall [36,37,38]	21 (13.6–28.4)	0.50	973.61 (970.13–976.75)	31.40 (7.80–63.0)	--
Total (mean)	--	--	--	38.49 (12.94–73.78)	--
Weighted average	151.37 (132–224)	0.48	982.86 (980.02–985.42)	--	--

**Table 2:** Material properties of three variants of silicone. Moduli of elasticity and the Poisson’s ratios were determined from the uniaxial tensile tests, and the densities were determined by measuring the weights of known volumes of the materials.

Material	Modulus of Elasticity, *E_100%_* (KPa)				Poisson’s Ratio, *µ*				Density, *ρ* (Kg/m^3^)
	Sample 1	Sample 2	Sample 3	Median	Sample 1	Sample 2	Sample 3	Median	
Dragon Skin FX-Pro	81.3	90.9	148.3	90.9	0.47	0.51	0.52	0.51	1120
Dragon Skin 10 NV	207.4	185.5	189.4	185.5	0.45	0.49	0.48	0.48	1030
Dragon Skin 10 Medium	189.4	182.7	196.1	189.4	0.41	0.46	0.46	0.46	1210

**Table 3:** Uterine wall tensions at different gestational ages and the corresponding elongations required along both axes (*X*, *Y*) simultaneously to create that tension in the membrane.

Property	Gestational Age (Week)		
	20	25	30
Maximum uterine wall tensile stress, mean (range) (KPa) [34]	11.9 (5.8–19.5)	16.3 (9.5–24.2)	22.8 (14.2–33.3)
Elongation necessary to apply the stress, mean (range) (mm)	0.033 *a* (0.017–0.059) *a*	0.046 *a* (0.017–0.059) *a*	0.0639 *a* (0.043–0.1) *a*

**Table 4:** Description of different “Kick Mode” available in the “Fetal Kick Simulator” software.

Kick Mode	Description
Single	Only one actuator operates in this mode.
Dual: Simultaneous	Two actuators operate simultaneously in this mode. The number of kicks for both the actuators must be equal, and they will start simultaneously at the beginning of each kick.
Dual: Consecutive	Actuators run consecutively one after another.
Dual: Random	Actuators start randomly based on the respective user-defined kick profile.

**Table 5:** Vibration characteristics of the testbed abdomen model. Ten sets of data were used to obtain the natural frequency of the abdomen model.

Property	Value
Natural frequency obtained from the accelerometer, (mean ± SD) (Hz)	18.70 ± 0.10
Natural frequency obtained from the acoustic sensor, (mean ± SD) (Hz)	18.75 ± 0.17
Damping ratio, *ζ*	0.80
Time constant, *τ* (ms)	6.40

**Table 6:** Comparison of the characteristics of real and simulated fetal kicks. Forty sets of measurements at different locations of the membrane were used to calculate the average kick reaction force for each wall displacement.

Kicking Entity	Uterine Wall Displacement, (Mean ± SD) (mm)	Kick Duration, (Mean ± SD) (s)	Kick Reaction, (Mean ± SD) (N)
20-week old fetus [30]	11.78 ± 4.72	2.65 ± 0.35	28.85 ± 1.88
30-week old fetus [30]	11.52 ± 1.47	2.95 ± 0.74	46.64 ± 5.30
Cylindrical probe, 10 mm diameter	10	1.63	14.84 ± 1.76
	15	2.45	33.15 ± 3.92
Hemispherical probe, 30 mm diameter	10	1.69	26.01 ± 1.66
	15	2.53	44.20 ± 2.37

## Supplementary Materials

The following is available online at https://www.mdpi.com/1424-8220/20/21/6020/s1. A video of the FM simulator in action.

## Data Accessibility

Software for the FM simulator and all the relevant data are available online at https://github.com/IncidentalCoder/Imperial_Kick_Simulator.git.
